# Multi-Omics Analysis Reveals the Dynamic Changes of RNA N*^6^*-Methyladenosine in Pear (*Pyrus bretschneideri*) Defense Responses to *Erwinia amylovora* Pathogen Infection

**DOI:** 10.3389/fmicb.2021.803512

**Published:** 2022-02-10

**Authors:** Chenyang Han, Feng Zhang, Xin Qiao, Yancun Zhao, Qinhai Qiao, Xiaosan Huang, Shaoling Zhang

**Affiliations:** ^1^State Key Laboratory of Crop Genetics and Germplasm Enhancement, College of Horticulture, Nanjing Agricultural University, Nanjing, China; ^2^Institute of Plant Protection, Jiangsu Academy of Agricultural Sciences, Nanjing, China

**Keywords:** transcriptomics, MeRIP-seq, plant defense, fire blight, mRNA stability, N^6^-methyladenosine (m^6^A)

## Abstract

N6-methylated adenine (m^6^A) is the most prevalent modification of mRNA methylation and can regulate many biological processes in plants, such as mRNA processing, development, and stress response. Some studies have increased our understanding of its various roles in model plants in recent years. Nevertheless, the distribution of m^6^A and the impact of m^6^A on the regulation of plant defense responses against pathogen inoculation are virtually unknown in pear. In this study, MeRIP-seq and RNA-seq data from healthy and inoculated plants were analyzed to assess the changes in the transcript levels and posttranscriptional modification of pear in response to the fire blight pathogen *Erwinia amylovora*. Following the analysis of 97,261 m^6^A peaks, we found that m^6^A preferred to modify duplicate genes rather than singleton genes and that m^6^A-methylated genes underwent stronger purifying selection. A total of 2,935 specific m^6^A sites were detected at the transcriptome level after inoculation, which may increase defense-related transcript abundance to enhance pear resistance. In addition, 1,850 transcripts were detected only in the mock-inoculated groups. The hypomethylated transcripts were mainly related to transcriptional regulation and various biological processes, such as chloroplast organization and sucrose biosynthetic processes. In addition, we found that the extent of m^6^A methylation was significantly positively correlated with the transcript level, suggesting a regulatory role for m^6^A in the plant response.

## Introduction

Posttranscriptional modification is an important posttranscriptional regulatory mechanism through which RNA transcripts can be ensured to work normally at any given time (Wang et al., 2018; Arribas-Hernandez and Brodersen, 2020). N6-methylated adenine (m^6^A) is the most prevalent modification in mRNA methylation that can be regulated by transcription, splicing, and translation, accounting for 80% of total RNA methylation modifications (Kierzek and Kierzek, 2003; Fu et al., 2014). Since m^6^A was first found in the 1970s, it has been widely identified in bacteria, viruses, plants, fungi, and mammals (Jia et al., 2013; Liu et al., 2014; Zheng et al., 2020). m^6^A modification is a dynamically reversible process regulated by a number of proteins, including methyltransferases (writers), demethylases (erasers), and m^6^A-binding proteins (readers), which act synergistically to regulate the abundance of m^6^A (Dominissini et al., 2012; Luo et al., 2014; Yue et al., 2019). Writers and erasers can bind the conserved consensus sequence RRACH (R = A or G; H (=A, U, or C) to add and remove m6A modification, and these modified RNAs eventually perform various functions by the binding of readers to m6A sites (Schwartz et al., 2013; Li et al., 2014; Parker et al., 2020). Following the discovery of the first m6A writer (METTL3) in mammals, a series of m6A-related enzymes have been found. METTL3 and METTL14 together form a heterodimer with the support of cofactors to induce m6A methylation (Bokar et al., 1994; Liu et al., 2014). The discovery of the first m6A eraser, obesity-associated protein (FTO), proves that RNA modification is dynamically reversible, but in recent studies, N6,2′-O-dimethyladenosine (m^6^A_m_) was proven to be the substrate of FTO (Jia et al., 2011; Mauer et al., 2017). The second identified m^6^A demethylase, ALKBH5, shows demethylation activity similar to that of FTO and is connected with cancer pathogenesis (Zheng et al., 2013). m^6^A readers exert a more specific regulatory function by binding m^6^A modification sites on RNA (Dominissini et al., 2012). RNA processing is also affected by m^6^A reader proteins; two kinds of m^6^A readers, YTHDF and YTHDC, can bind to the m^6^A sites in mRNA to implement the biological function of methylation modifications (Du et al., 2016; Patil et al., 2018; Scutenaire et al., 2018). It has now become clear that this reversible posttranscriptional modification is indispensable for gene regulation.

At present, research on plant m^6^A is mainly in mammalian systems and rarely in plants. Previously, several studies have investigated the role of m^6^A in mRNA stability, plant growth and development, and stress processes (Arribas-Hernandez and Brodersen, 2020). Some evidence suggests that plants display a different m^6^A modification pattern than animals. In *Arabidopsis*, MTA (METTL3 human homologue protein), MTB (METTL14 human homologue protein), FIP37 (WTAP human homologue protein), VIRILIZER (KIAA1429 human homologue protein), and HAKAI (HAKAI human homologue protein) are considered the five components of m^6^A writers (Liu et al., 2014; Kan et al., 2017; Ruzicka et al., 2017; Arribas-Hernandez et al., 2018; Scutenaire et al., 2018). The lack of MTA and MTB results in a decrease in m^6^A-modified mRNAs. Another member of the core m^6^A methylation family, the MTA-interacting protein FIP37, plays an important role in embryonic development and shoot stem cell fate (Vespa et al., 2004; Anderson et al., 2018). Inhibition of the expression of VIRILIZER and HAKAI resulted in a decrease in the level of m^6^A in *Arabidopsis* (Ruzicka et al., 2017). However, the number of m^6^A enzymes found to date in plants is small relative to the number in animals. Complex m^6^A modifications in plants suggest that some components of the m^6^A system are undetected, and the major m^6^A eraser FTO in mammals has not been found in plants (Hofmann, 2017; Yue et al., 2019). To date, it has been found that members of the ALKB family could be m^6^A erasers in plants. ALKBH9B and ALKBH10B are considered to be important components involved in the demethylation of *Arabidopsis*, and they were shown to revert m^6^A to adenosine (Duan et al., 2017; Martinez-Perez et al., 2017). Among the most important m^6^A readers, the ECT family in *Arabidopsis* contains a YTH domain to recognize m^6^A sites (Arribas-Hernandez et al., 2018). The binding ability of ECT2 to m^6^A depends on a tritryptophan pocket in plants, and it also improves the stability of m^6^A-methylated RNAs transcribed from genes related to trichome morphogenesis. In addition to RRACH, plants possess a specific consensus motif, URUAY (R (=A or G; Y (=U or C), which can be recognized by the m^6^A reader ECT2 (Scutenaire et al., 2018; Wei et al., 2018).

Methylated RNA immunoprecipitation with high-throughput sequencing (MeRIP-Seq) provides an effective method to further analyze the function of m^6^A modifications in plants (Cooper, 2012; Luo et al., 2014). By this method, it has been found that m^6^A can be selectively added to salt response proteins under salt stress (Anderson et al., 2018; Zheng et al., 2021). Although these studies have demonstrated the importance of m^6^A in plant growth and stress responses, whether m^6^A modifications can stabilize mRNAs is still controversial (Anderson et al., 2018).

Fire blight caused by *Erwinia amylovora* is one of the most damaging diseases on pear and other Rosaceae (Born et al., 2017; Schachterle and Sundin, 2019). Understanding the mechanistic basis of this host–pathogen interaction is imperative for elucidating the pathogenesis of fire blight. However, the underlying molecular mechanism of the resistance and susceptibility of pear to *Erwinia amylovora* is largely unknown (McNally et al., 2012). In this study, we focused on the changes in m^6^A modifications and mRNA levels in pear after fire blight inoculation by transcriptome sequencing (RNA-seq) and MeRIP-Seq. The expression changes at different points during inoculation revealed that plants activate immune responses rapidly, as early as 3 h after inoculation, and substantial changes were detected in gene expression levels. After a period of time, the plant immune response entered a stable period and then changed again 3 days later. These transcription-level changes attracted our interest. Next, we focused on the regulation of gene transcription and mRNA modification in pear throughout the defense processes. m^6^A methylation was shown to be dynamic and reversible through MeRIP-Seq. We found that m^6^A modifications can be selectively added to defense-related genes to increase expression abundance after fire blight inoculation. In addition, m6A modification was removed from a considerable number of transcripts during inoculation, including DNA-binding transcription factors and genes related to transcriptional regulation. Finally, we further confirmed that gene expression is positively correlated with m^6^A abundance.

## Materials and Methods

### Samples Collected for RNA-Seq and Methylated RNA Immunoprecipitation With High-Throughput Sequencing

Tissue-cultured plantlets of pear were cultivated in State Key Laboratory of Crop Genetics and Germplasm Enhancement in Nanjing Agricultural University. The fire blight pathogen was lyophilized and stored at −80°C in a freezer. Before their use, they were streaked on NA agar plates and cultured in liquid LB medium at 28°C for 16 h. Plant samples were collected at 3, 12, 24, 48, and 72 h post-inoculation and uninoculated seedlings were used as controls (mock); each time point had three duplications (three plants per replicate; 18 samples in total). The plants were grown in MS medium in growth chambers with 16 h of light and 8 h of darkness at 26°C with a relative humidity of 80%. The leaves were immediately frozen in liquid nitrogen and stored at −80°C until further use. These samples were used for RNA-seq and the same samples (mock, 12 HPI) were used to for MeRIP-Seq.

### RNA-Seq and Data Analysis

RNA extraction and sequencing were done by Novogene Corporation (Nanjing, China). Total RNA was isolated using the Plant RNA Isolation Kit (Macrogene). RNA purity was checked using the NanoPhotometer spectrophotometer (IMPLEN, CA, United States). RNA concentration was measured using Qubit RNA Assay Kit in Qubit 2.0 Flurometer (Life Technologies, CA, United States). RNA integrity was assessed using the RNA Nano 6000 Assay Kit of the Bioanalyzer 2100 system (Agilent Technologies, CA, United States). A total amount of 3 μg RNA per sample was used as input material for the RNA sample preparations. Sequencing libraries were generated using NEBNext Ultra RNA Library Prep Kit for Illumina (NEB, United States). The library preparations were sequenced on an Illumina Hiseq platform and 125 bp/150 bp paired-end reads were generated.

Raw data (raw reads) of fastq format were firstly processed through in-house perl scripts and clean reads were obtained by removing reads containing adapter, reads containing ploy-N, and low-quality reads from raw data. For each sample, Q30, Q20, and GC content were calculated; all the downstream analyses were based on the clean data. The clean reads were aligned to the Chinese white pear genome (cv. “Dangshansuli”) using HISAT2 (Kim et al., 2015). The read counts of each sample were obtained by FeatureCounts (Liao et al., 2013). Finally, read counts were normalized to tags per million (TPM) by Tbtools (Chen et al., 2020). Correlation analysis of m^6^A writers, erasers, and readers under drought treatment and cold treatment was calculated using cor (a function in R).

### Differential Gene Expression and Enrichment Analysis

The read counts were used to perform differential gene expression analysis with DESeq2 R package (v1.30.1). GO (Gene Ontology) and KEGG (Kyoto Encyclopedia of Genes and Genomes) pathway enrichment analysis was performed with KOBAS software (Xie et al., 2011)^1^.

### Methylated RNA Immunoprecipitation With High-Throughput Sequencing and Data Analysis

MeRIP-Seq was performed by Cloudseq Biotech Inc. (Shanghai, China) according to the published procedure with slight modifications. Briefly, fragmented RNA was incubated with anti-m^6^A polyclonal antibody (Synaptic Systems, 202003) in IPP buffer for 2 h at 4°C. The mixture was then immunoprecipitated by incubation with protein-A beads (Thermo Fisher Scientific) at 4°C for an additional 2 h. Then, bound RNA was eluted from the beads with N^6^-methyladenosine (BERRY and ASSOCIATES, PR3732) in IPP buffer and then extracted with Trizol reagent (Thermo Fisher Scientific) by following the manufacturer’s instruction. Purified RNA was used for RNA-seq library generation with NEBNext Ultra RNA Library Prep Kit (NEB). Both the input sample without immunoprecipitation and the m^6^A IP samples were subjected to 150 bp paired-end sequencing on Illumina HiSeq sequencer.

The raw reads were retrieved as clean reads using the Cutadapt software tool (v1.9.3). The clean reads of input and IP libraries were mapped to genome by HISAT2 (Kim et al., 2015). Methylated sites on RNAs (peaks) were identified by MACS software (Zhang et al., 2008). Differentially methylated sites were identified by diffReps (Shen et al., 2013). Consensus sequence motifs enriched in m^6^A peaks were identified by MEME^2^. The visualization of the m^6^A abundance was present by Integrative Genomics Viewer (IGV). The circos plot of m^6^A peaks and DEPs across chromosomes were generated using the circos package in R.

### Duplicated Gene Pairs Identification and K_a_/K_s_ Calculation

A gene duplication analysis was conducted in pear genome and m^6^A genes, and the method was proposed by Qiao et al. (2019) The DupGen_finder pipeline was used to identify the different modes of duplicated gene pairs^3^, and the genome of *Vitis vinifera* was used as outgroup (Jaillon et al., 2007). To determine the duplication type of each gene, we used DupGen_finder-unique to assign the duplicate genes to a unique mode (Qiao et al., 2019). The priority of the duplicate genes is as follows: WGD > tandem > proximal > transposed > dispersed. The K_a_/K_s_ ratio was calculated using the calculate_Ka_Ks_pipelin (Wang et al., 2010).

### LC-MS/MS Quantification of RNA Modification in Total RNA

LC-MS/MS analysis refers to the means of Adams et al. (2020) with minor modifications. Total RNA (1 μg) was digested by buffer 1 (300 mM CH_3_COONa, 2,800 mM NaCl, 10 mM ZnSO_4_, pH 4.6), 1 μl of the S1 nuclease (180 U/μl) was added, and the samples were incubated at 37°C for 4 h. Then 10 μl buffer 2 (500 mM Tris–HCl, 10 mM MgCl_2_, pH 9.0) was added, followed by the addition of 5 μl Venom phosphodiesterase I (0.002 U/μl) and 1 μl alkaline phosphatase (30 U/μl). The samples were incubated again at 37°C for 2 h. The nucleosides were separated by reverse phase high-performance liquid chromatography on an Agilent C18 column, coupled with mass spectrometry detection using AB SCIEX QTRAP 5500. The m^6^A levels were calculated as the ratio of m^6^A to A based on the calibrated concentrations according to the standard curve obtained from pure nucleoside standards running with the same batch of samples.

### qRT-PCR and m^6^A-IP-qPCR

For qRT-PCR, total RNA extraction and the synthesis of cDNA were according to the instructions of RNA kit (Tiangen, Beijing, China) and PrimeScript RT reagent kit (Trans Gen). qRT-PCR was performed using a LightCycler 480 SYBR-GREEN I Master (Roche, United States), and tubulin (Tub) is used as the reference genes. m^6^A-IP–qPCR was performed as previously described (Dominissini et al., 2013) immediately after m^6^A-IP enrichment. The same amount of the concentrated IP RNA or input RNA from each sample was used for the cDNA library. The relative m^6^A enrichment in genes were calculated by the m^6^A levels (m^6^A IP) normalized using the input of each gene. Relative levels of genes were calculated using the 2^–ΔΔCt^ method. The primers are shown in Supplementary Table 12.

## Results

### Transcriptome Sequencing Revealed Rapid Transcriptional Changes in the Pear Response in the Early Stage After Fire Blight Inoculation

**FIGURE 1:**
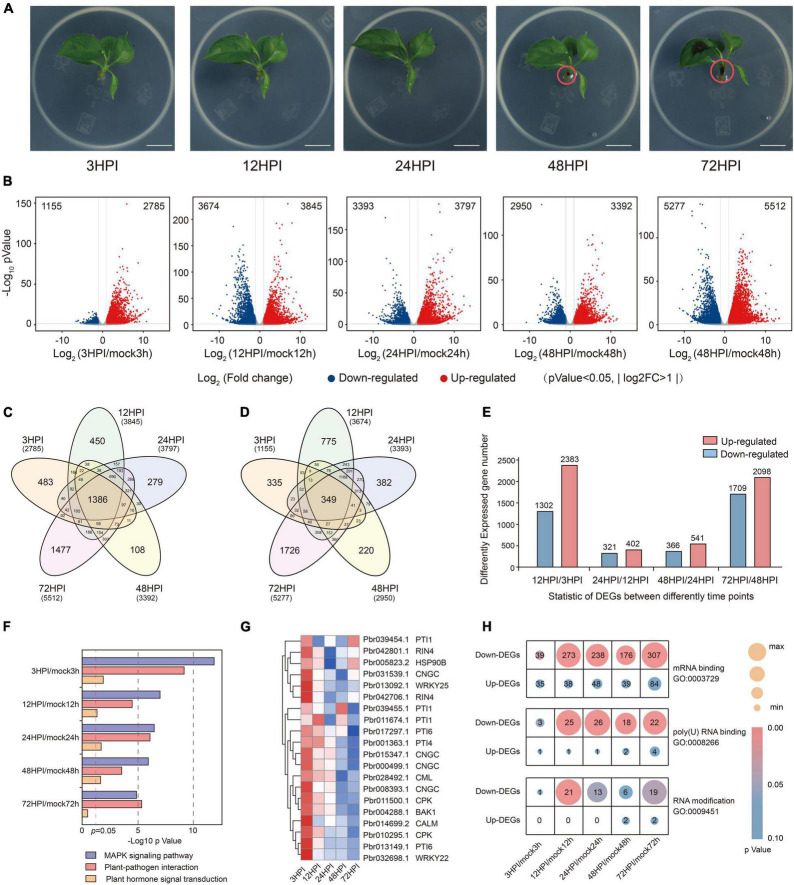
Significant changes in transcription levels after fire blight inoculation. **(A)** Images of pear seedlings after fire blight inoculation at 3 HPI, 12 HPI, 24 HPI, 48 HPI, and 72 HPI. Scale bar = 1 cm. **(B)** Volcano plot of DEGs (differentially expressed genes) between *Erwinia amylovora*–inoculated and mock-inoculated at different time points. **(C,D)** Venn diagram showing the number of DGEs identified between *Erwinia amylovora*–inoculated and mock-inoculated at different time points. **(C)** Upregulation. **(D)** Downregulation. **(E)** The bars indicate the number of DEGs between samples from adjacent time points, red indicates upregulation, and blue indicates downregulation. **(F)** KEGG pathway analysis of upregulated genes in plant hormone signal transduction, plant–pathogen interaction, and MAPK signaling pathway. **(G)** Expression profiles of DEGs related with plant–pathogen interaction pathway. **(H)** Gene number of DEGs involved in GO term mRNA binding (GO:0003723), poly(U) RNA binding (GO:0008266), and RNA modification (GO: 0009451).

To begin studying the possibility that pear responds to fire blight, we inoculated *Erwinia amylovora* into pear seedlings, and LB liquid medium was inoculated to serve as a mock inoculation. The seedlings were observed for 72 h post-inoculation (HPI). Disease symptoms developed at 12 HPI, and mild lesions were observed at the inoculation site (Figure 1A). Notably, before significant lesions were found in leaves, the stems were infected and melanized at 48 HPI (red circle; Figure 1A). The leaves developed clear symptoms of inoculation at 72 HPI; subsequently, the stem base became completely black, and the entire plant wilted. Samples were collected at 0 (preinoculation), 3, 12, 24, 48, and 72 HPI for transcriptome sequencing, and each time point contained three replicates. From these samples, approximately 1.62 billion raw reads were produced. Following filtering, 1.59 million high-quality-filtered (clean) reads proceeded to the next step of the study, and the average read count for each sample ranged from approximately 40 to 63 million. The resulting clean reads were aligned against the Chinese white pear genome (Wu et al., 2013) (cv. “Dangshansuli”), with mapping rates ranging from 76.64 to 79.22% (Supplementary Table 1).

Differential expression results were generated using DESeq2, and gene expression was quantified using transcripts per million (TPM). Genes with expression | fold change| > 2 (*p* < 0.05) were considered to be differentially expressed compared with the previous time point, and they were visualized through volcano plots (Figure 1B). In total, 3,940 (2,785 were upregulated and 1,155 were downregulated), 7,519 (3,674 were upregulated and 3,845 were downregulated), 7,190 (3,393 were upregulated and 3,797 were downregulated), 6,342 (2,950 were upregulated and 3,392 were downregulated), and 10,789 (5,277 were upregulated and 5,512 were downregulated) differentially expressed genes (DEGs) were identified at 3, 12, 24, 48, and 72 HPI (Figure 1B). We next compared DEGs among different time points; indeed, 72 HPI had the largest number of genes among all of the DEGs in the mock- and Ea-inoculated groups (Figures 1C,D). This result suggested that relatively little change occurred in the initial stage of inoculation. To test this hypothesis, the same method was performed to determine which pairs of adjacent time points differed significantly from each other by DESeq2 (Figure 1E). Analysis of these DEGs revealed a significant difference after inoculation, and these changes were particularly pronounced during early stages of fire blight inoculation (within 12 h). However, changes were less apparent from 12 to 48 HPI, after which they started to rise again at 72 HPI. To learn more about the changes during plant defense, we focused on the three KEGG pathways associated with plant defense, including the MAPK signaling pathway, plant–pathogen interaction, and plant hormone signal transduction. It was found that the enrichment of the MAPK signaling pathway and plant–pathogen interaction decreased with time, and the gene expression level declined over time (Figures 1F,G), possibly indicating that the defense response to fire blight is initially intense and wanes over time. The identified DEGs were subjected to gene ontology (GO) analysis (Supplementary Table 2), and the GO terms with high fold enrichment in upregulated DEGs at 3 HPI were “defense response to bacterium (GO: 0042742)” and “defense response (GO: 0006925),” which indicated that the defense response of plants was very rapid, occurring within 3 h. At consecutive time points, GO analysis indicated that the enriched GO terms in upregulated mRNAs were mainly associated with the defense response. Studies have reported that fire blight inoculation can induce an increase in jasmonic acid levels in apple (Kamber et al., 2016). We found significant enrichment of jasmonic acid–related GO terms, including jasmonic acid biosynthetic process (GO:0009695) and response to jasmonic acid (GO:0009753), in upregulated DEGs during the entire inoculation process. Interestingly, the number of DEGs decreased significantly from 12 to 48 HPI. Therefore, we hypothesized that the defense responses of plants reached a steady state between 12 and 48 HPI. In a recent study, it was reported that m^6^A has an impact on the resistance of apple to powdery mildew (Guo et al., 2021). Subsequently, we counted the RNA-related genes annotated by the GO terms “mRNA binding (GO:0003729),” “poly(U) RNA binding (GO:0008266),” and “RNA modification (GO:0009451)” in DEGs and found that they were enriched, especially after 12 HPI (Figure 1H). We hypothesize that fire blight inoculation might affect the transcription and stability of the mRNA and that plants might control mRNA levels by regulating the modification of mRNA. These results indicate that plants responded very quickly to fire blight inoculation. After a period of stability, the response of plants again became apparent with increasing inoculation severity.

### The Expression Pattern Changes of m^6^A Regulators Suggest That m^6^A Plays a Role During Fire Blight Inoculation

**FIGURE 2:**
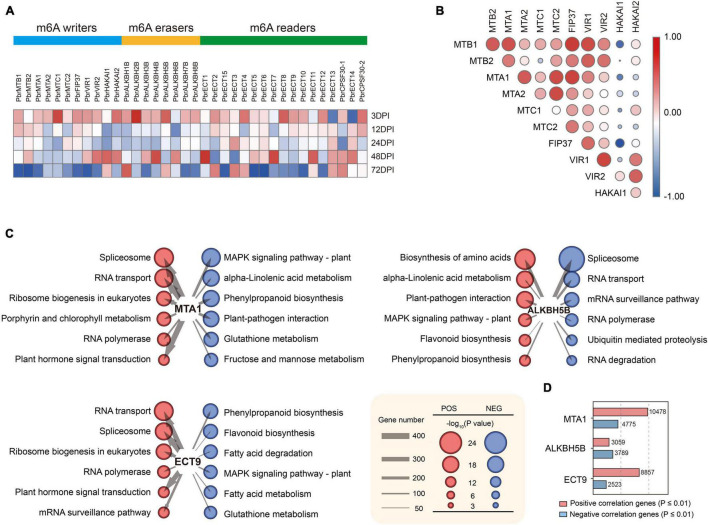
Expression alterations of m^6^A writers, erasers, and readers in pear after fire blight inoculation. **(A)** Heat map of RNA-Seq expression of m^6^A regulators. Color scale of the dendrogram represents the scale value of TPM, normalized expression data expressed as log2 (TPM+1) values. **(B)** Correlation among the expression of m^6^A regulators. **(C)** Network diagram demonstrating the correlation between m^6^A regulators and signaling pathways. Red represents a positive correlation, and blue represents a negative correlation. The size of the nodes corresponds to the number of genes enriched in the entry. The size of the circles corresponds to -log_10_(*p*-value) of KEGG pathways. **(D)** Number of genes that significantly correlated with MTA1, ALKBH5B, and ECT9.

Gene expression is regulated by multiple posttranscriptional modifications, such as m^6^A (Shi et al., 2017). The m^6^A modification is mediated by the concerted action of m^6^A writers, erasers, and readers. We used the same methodology presented by Yue et al. (2019) to identify the m^6^A regulators in pear, including 11 writers, 8 erasers, and 17 readers (Supplementary Table 3). The number of m^6^A regulators was much greater than that in *Arabidopsis* due to recent whole-genome duplications (WGD) in pear. Notably, the m^6^A writers (MTA, MTB, MTC, FIP37, VIR, HAKAI) in pear were almost double those in *Arabidopsis*. These duplicate genes have survived WGD, and it is unclear whether they can regulate m^6^A methylation through different molecular regulatory mechanisms. We analyzed the K_a_ (number of substitutions per non-synonymous site), K_s_ (number of substitutions per synonymous site), and K_a_/K_s_ values to detect the selection pressure acting on m^6^A regulators. The K_a_/K_s_ values of all the m^6^A regulators were less than one, indicating that these genes evolved through purifying selection (Supplementary Table 4). We further examined whether fire blight inoculation affects the expression of m^6^A regulators, and the RNA levels were tested by qRT-PCR. The expression pattern of m^6^A writers was significantly downregulated after inoculation (Figure 2A and Supplementary Figure 1A). In addition, we found high correlations among these m^6^A regulators, such as PbrMTA1 and PbrFIP37 (Figure 2B and Supplementary Figure 1B).

### m^6^A Regulators Were Significantly Associated With RNA- and Defense-Related Pathways

To further study which signaling pathways are associated with m^6^A regulators, we performed a coexpression network analysis to illuminate the collaboration between m^6^A regulators and bulk mRNA data in 30 transcriptome samples. Pearson correlations between behavioral test scores were calculated using the corr.test. Here, MTA1, ALKBH5B, and ECT9 showed significant correlations (*p*-value ≤ 0.01) with a large set of genes (Figure 2D), and these genes were then employed for KEGG enrichment analysis (Figure 2C). We found that the expression of m^6^A regulators was correlated with multiple RNA-related signaling pathways, such as spliceosome, RNA transport, and RNA polymerase. The results revealed that the RNA-related signaling pathways were negatively correlated with the m^6^A writer MTA but positively correlated with the m^6^A eraser ALKBH5B and the m^6^A reader ECT9. Notably, MTA and ALKBH5B were also associated with plant–pathogen interactions, MAPK signaling pathways, alpha-linolenic acid metabolism, and flavonoid biosynthesis. In addition, protein–protein interaction (PPI) analysis showed frequent interactions among these writers, erasers, and readers (Supplementary Figure 1C). Among them, m^6^A writers had the highest number of interactions. Taken together, these results suggest that the cross-talk among the m^6^A regulators plays critical roles during fire blight inoculation.

### Transcriptome-Wide m^6^A Methylation Profiles in Pear

Based on the expression changes of m^6^A regulator levels in the progression of fire blight inoculation, we performed MeRIP-seq to profile a transcriptome-wide m^6^A map of pear. This series included mock-inoculated control plants (mock) and fire blight–infected plants (12 HPI), and each group had three biological repeats (Supplementary Table 5). After alignment to the Chinese white pear reference genome (cv. “Dangshansuli”), a total of 97,261 m^6^A peaks were identified in mock plus treatment plants using MACS2 (Supplementary Table 6). At the genome level, m^6^A modifications were not evenly distributed across each chromosome, and the Circos plot showed good repeatability among sequencing samples (Supplementary Figures 2A,B). The m^6^A-modified gene transcripts consistently detected in three biological replicates were considered high-confidence genes; in total, 10,544 mock-specific genes and 10,729 12 HPI-specific genes were used in subsequent studies. We found that m^6^A modifications were highly enriched around the start and stop codons in pear (Figure 4A), consistent with the m^6^A distribution in *Arabidopsis* and tomato (Wan et al., 2015; Zhou et al., 2019). We detected the enrichment of m^6^A modifications in gene transcripts; most (74.91%) contained only a single m^6^A peak, 21.22% contained two m^6^A peaks, 3.21% exhibited three peaks, and 0.67% exhibited more than three peaks (Figure 4B), demonstrating that m^6^A modifications are highly conserved in pear. Reads were visualized using IGV to check repeatability among sequencing samples, and the results showed good repeatability among each sample (Figure 4C). MEME was used to identify sequence motifs enriched within the m^6^A peaks, and previously established motifs RRACH and UGUAYY were identified in pear as in *Arabidopsis* (Bailey et al., 2006; Figure 4D). We also examined the distribution of m^6^A modifications in inoculated plants (Supplementary Figure 3). The results showed that fire blight inoculation was unlikely to have a high impact on the m^6^A modification position in transcripts, and m^6^A modifications were enriched near the start and stop codons.

**FIGURE 3:**
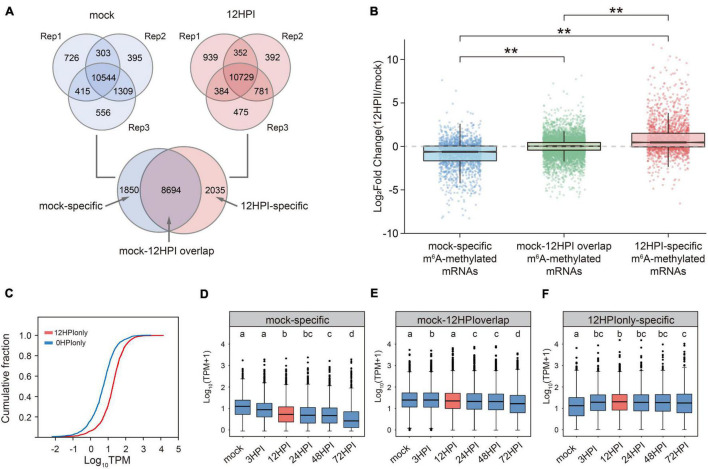
Fire blight inoculation induces changes in transcriptome-wide m^6^A modification, resulting in the increase of RNA abundance of m^6^A modified transcripts. **(A)** Overlap of m^6^A-modified genes among three replicates of mock (blue circles) and 12 HPI (red circles), the intersection of replicates indicates high-confidence m^6^A-modified genes. The subset of 0 HAI and 12 HAI indicates specifically m^6^A-modified genes before and after blight inoculation. **(B)** Relative abundance of m^6^A-modified gene transcripts in mock to 12 HPI contain mock-specific (blue box), 12 HPI–specific (red box), and mock-12 HPI overlap (green box). *p < 0.05; **p < 0.01. **(C)** Cumulative distribution function (CDF) of the relative abundance (12 HPI divided by mock) of transcripts in mock-specific m^6^A-modified genes and 12 HPI–specific m^6^A-modified genes compared using the Wilcoxon *t*-test. **(D–F)** Expression pattern of m^6^A-modified genes at different time points after inoculation.

**FIGURE 4:**
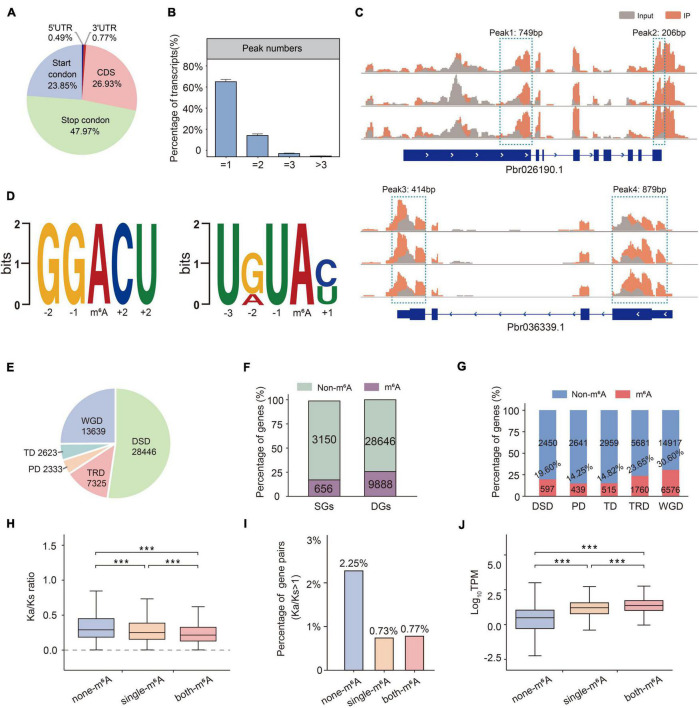
Overview of m^6^A methylation profiles in pear. **(A)** Percentage of total m^6^A peaks located throughout the regions of mRNA transcript. **(B)** Proportions of different m^6^A peak numbers in m^6^A-modified transcripts. Error bars represent the SD of three biological repeats. **(C)** Two examples of m^6^A-modified transcripts each containing two peaks at start codon, stop codon, and CDS (coding sequence). **(D)** Sequence motif identified by MEME. **(E)** The number of gene pairs derived from five duplication types in pear. **(F)** Comparison of ratios of m^6^A genes and non-m^6^A genes in SGs and DGs. **(G)** Comparison of ratios of m^6^A genes and non-m^6^A genes in five duplication S types. **(H)** K_a_/K_s_ ratio of non-m^6^A gene pairs (blue box), single gene pairs (orange box), and both-m^6^A gene pairs (red box) in WGD. **(I)** The percentages of gene pairs showing K_a_/K_s_> 1 in WGD. **(J)** Expression divergence among duplicate genes derived from non-m^6^A, single-m^6^A, and both-m^6^A in WGD.

The pear genome was confirmed to have undergone at least two WGD events (4 dTv of ∼0.08; 4 dTv of ∼0.5), which provided us with a suitable system to study the effect of m^6^A on gene duplication (Wu et al., 2013). Here, we reviewed the evolutionary process of pear, and the duplicated gene pairs were classified into five categories by DupGen_finder (Qiao et al., 2019): DSD (dispersed duplication), TD (tandem duplication), PD (proximal duplication), TRD (transposed duplication), and WGD (whole-genome duplication) (Figure 4E). Of note, by examining the m^6^A modification on duplicated genes (DG) and singleton genes (SG), we found that m^6^A preferred DGs (25.66%) over SGs (17.23%) (Figure 4F). These genes that survived in duplicate from WGD had higher m^6^A methylation rates than the others. Then, we detected m^6^A-modified genes in pear, and they were assigned to DSD, TD, PD, TRD, and WGD (Figure 4G and Supplementary Table 7). Overall, ∼31% WGD and ∼24% TRD genes were methylated in pear. We reasoned that the genes generated by WGD and TRD can maintain m^6^A modification better than other duplication types. Based on our previous study, TRD may be important for plants to adapt to dramatic environmental changes (Qiao et al., 2019). Another study showed that m^6^A prefers actively transcribed genes (Zheng et al., 2020), and we hypothesize that epigenetic variation after gene duplication may lead to a greater activating function in plants. To investigate the coevolutionary consequences of gene duplication events and m6A modification in pear, we classified the gene pairs according to the m^6^A modification. The gene pairs were divided into three categories: gene pairs within two m^6^A-modified genes (both-m^6^A), gene pairs within a single m^6^A-modified gene (single-m^6^A), and gene pairs with no m^6^A-modified genes (non-m^6^A). Surprisingly, we found that both m^6^A gene pairs had the highest K_a_/K_s_ ratios (Figures 4H,I) and the lowest expression (Figure 4J) in WGD. These findings suggest that non-m^6^A genes experience stronger positive selection and that m^6^A may affect the evolution of new biological functions.

### Differentially m^6^A-Modified Genes in Mock- and Pathogen-Inoculated Plants

To study the effect of m^6^A during plant defense, we detected the overall m6A methylation level by LC-MS/MS, but there was no significant difference between mock and 12 HPI (Supplementary Figure 5A). We next characterized the m^6^A-modified genes in mock-inoculated plants and 12 HPI plants. We identified 10,544 and 10,729 high-confidence genes in the mock and 12 HPI groups using MACS (Zhang et al., 2008; Figure 3A). Based on these results, 1,850 and 2,035 m^6^A-modified genes were unique to the mock and 12 HPI groups, respectively.

We then compared the relative abundance (log2[12 HPI mRNA-seq TPM divided by mock mRNA-seq TPM]) during fire blight inoculation for mRNAs with mock-specific, 12 HPI–specific, and mock-12 HPI overlapping m^6^A peaks (Figure 3B). We found a significant difference (*p*-value < 0.001; Wilcoxon *t*-test) in the relative abundance between mock-specific m^6^A-modified mRNAs and 12 HPI–specific m^6^A-modified mRNAs from 0 to 12 h (Figures 3B,C). In fact, mock-specific m^6^A-modified mRNAs decreased in abundance with the loss of m^6^A modification (blue box; median < 0), and 12 HPI–specific m^6^A-modified mRNAs increased in abundance with the addition of m^6^A modifications (red box; median > 0). The other transcripts were slightly increased in abundance (green box; median > 0; Figure 3B). The box plots also indicated that the data distributions were concentrated, particularly the transcripts without modification changes. We next compared the gene expression patterns of these three classes of mRNAs at more time points (Figures 3D–F). These results show that the expression level of mock-specific m^6^A-modified mRNAs had a decreasing trend (Figure 3D), whereas 12 HPI-specific m^6^A-modified mRNAs had an increasing trend (Figure 3F), while the other mRNAs remained flat (Figure 3E).

**FIGURE 5:**
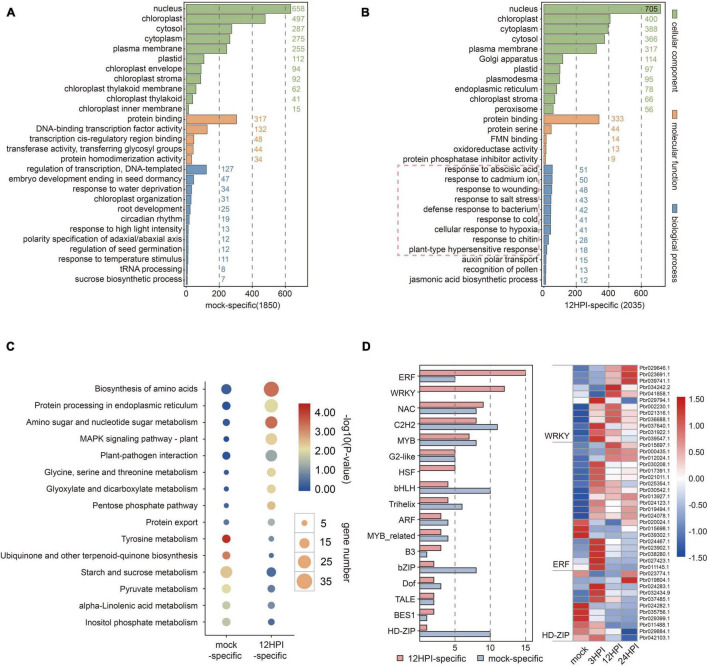
Functional analysis of mock-specific and 12 HPI-specific m^6^A-modified genes. **(A,B)** Gene Ontology (GO) enrichment analysis of mock-specific and 12 HPI–specific m^6^A-modified genes. **(C)** KEGG pathway enrichment analysis of mock-specific and 12 HPI–specific m^6^A-modified genes. **(D)** Distribution and expression analysis of transcription factors (TFs) involved in mock and 12 HPI.

To characterize the mock-specific m^6^A-modified mRNAs and 12 HPI–specific m^6^A-modified mRNAs, we performed GO and KEGG pathway analyses on these two classes of mRNAs (Supplementary Tables 8, 9). We found that m^6^A modification was added to the genes encoding proteins involved in the defense response to bacteria, cellular response to hypoxia, and response to wounding after fire blight inoculation (Figure 5A). Conversely, genes with mock-specific m^6^A peaks were enriched for more general terms associated with transcriptional regulation, such as DNA-binding transcription factor activity, regulation of transcription, and DNA templating (Figure 5B). We speculated that m^6^A selectively adds or removes m^6^A modification to regulate the plant defense response. KEGG enrichment results also indicated that some genes involved in metabolic processes lost m^6^A modification, while some associated with defense response were modified after inoculation (Figure 5C). After that, we counted the number of transcription factors (TFs) for mRNAs with mock-specific m^6^A peaks and 12 HPI–specific m^6^A peaks (Figure 5D). There were differences in the number of TFs between the specific mock- and 12 HPI–specific genes, such as ERF, bZIP, and HD-ZIP. A remarkable finding is that higher numbers of WRKY TFs were found in genes with 12 HPI–specific m^6^A peaks but not in mock-specific genes. WRKY TFs are conventionally thought to play important roles in the regulation of plant immunity. The aforementioned results show that m^6^A can selectively remove or add m^6^A modification to specific transcripts to regulate mRNA abundance, and plants can secure themselves via this defense mechanism.

### Correlation Analysis of Gene Expression Level and m^6^A Level

**FIGURE 6:**
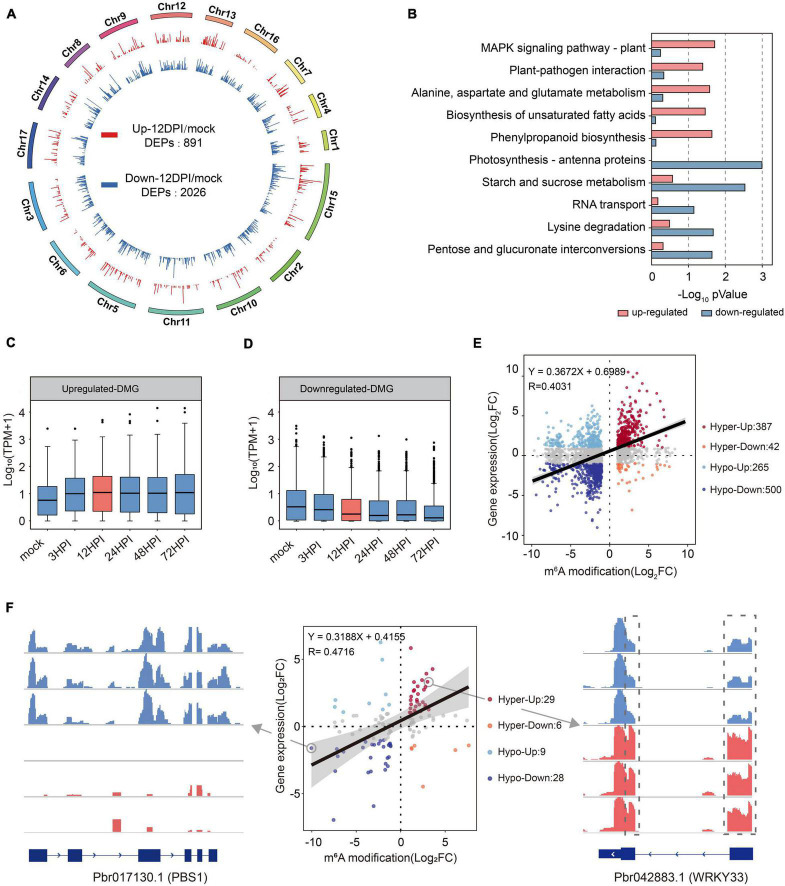
Association analysis of MeRIP-seq and RNA-seq reveals that m^6^A-modification results in the increase of transcript abundance. **(A)** Circos plots of pear genome showing the relative abundance of m^6^A modification across 17 chromosomes. Upregulated methylated peaks are shown in red and downregulated methylated peaks are shown in blue. The kurtosis represents fold change of m^6^A abundance. **(B)** KEGG pathway enrichment analysis of upregulated and downregulated methylated genes. **(C)** Distribution of genes with a significant change in both the m^6^A level and the gene expression level in mock compared with 12 HPI. The linear regression equations and *R*^2^ are shown on the graph. **(D,E)** Expression pattern of upregulated and downregulated methylated genes at different time points after inoculation. **(F)** Distribution of genes in plant–pathogen interaction KEGG pathway. Two genes were selected to present the browser views for m^6^A modification.

To further verify the relationship between gene expression patterns and m^6^A modifications, we localized differentially enriched peaks (DEPs) on a genome-wide scale using Diffreps (Shen et al., 2013). In total, 891 upregulated DEPs (red; fold change ≥ 2; *p* < 0.01) and 2,026 downregulated DEPs (blue; fold change ≤ 2; *p* < 0.01) were identified across 17 chromosomes (Figure 6A). These DEPs were mapped to 859 (upregulated) and 1,961 (downregulated) genes (differentially methylated genes; DMGs). Subsequently, m^6^A-upregulated genes and m^6^A-downregulated genes were subjected to GO and KEGG pathway analyses (Figure 6B and Supplementary Tables 10, 11). Similar to our previous results, m^6^A-upregulated genes focused on the defense response, and m^6^A-downregulated genes focused on transcription regulation and some plant life activities.

The conjoint analysis of the MeRIP-Seq and transcriptome data showed that the expression of genes with upregulated DEPs was significantly upregulated (Figure 6C). Conversely, the expression patterns of genes with downregulated DEPs were downregulated (Figure 6D). To further determine the relationship between gene expression and m^6^A abundance, the mock and 12 HPI RNA-seq data were selected for subsequent analysis. A total of 1,449 genes (| fold change| ≥ 2) were considered significantly different in their expression patterns (Figure 6E). Among these m^6^A-upregulated genes, 387 had increased mRNA levels (Hyperup), whereas 42 had reduced levels (Hyperdown). In addition, 500 had reduced mRNA levels (Hypo-down), whereas 265 had increased levels (Hypo-up) among m^6^A-downregulated genes. We observed a positive correlation between m^6^A abundance and mRNA levels (Pearson r correlation test; *R* = 0.4031; *p* < 0.01). To validate differential m^6^A abundance, we selected 98 genes involved in “plant–pathogen interaction” (ko: 04626) and verified the presence of m^6^A changes within WRKY transcription factors (Pbr042883.1) and serine/threonine kinases (Pbr017130.1) (Figure 6F). Eight genes were selected to validate the microarray results by qRT-PCR and m^6^A-IP-qPCR; we found that most of the genes were upregulated in methylation level and were also upregulated at the transcript level (Supplementary Figures 5B,C).

**FIGURE 7:**
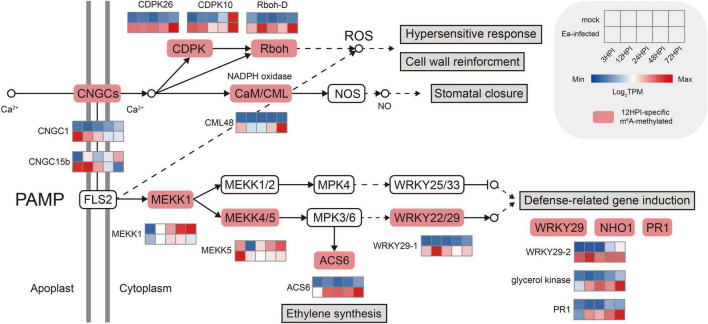
Visualization of pattern-triggered immunity (PTI) after fire blight inoculation. The red boxes indicate 12 HPI–specific m^6^A-methylated genes. The heatmap represents the expression patterns of the mock-inoculated plants and *Erwinia amylovora*–inoculated plants.

To further investigate the role of m^6^A in plant immunity, we concentrated on pathogen−associated molecular pattern (PAMP)–triggered immunity (PTI) genes (Figure 7). Among them, most PTI-responsive genes were modified after fire blight inoculation, including genes involved in signaling and systemic acquired resistance (SAR). In addition, their expression levels in pathogen-inoculated plants were significantly higher than those in mock-inoculated plants.

## Discussion

In this work, we investigated the impact of m^6^A methylation on plants under biotic stress. This is the first study that has used MeRIP-seq to study pear disease resistance. In addition, we also examined the association between m^6^A and gene duplication events. Our analysis provides important insights into the role of posttranscriptional modifications in plant defense and will be relevant for molecular breeding strategies for pear. Although m^6^A methylation in the plant response to abiotic stress has been preliminarily studied, only a few reports have focused on biotic stress. In a study of *N. tabacum* infected with tobacco mosaic virus, a decrease in the levels of m^6^A was detected after inoculation (Li et al., 2018). However, there is still a lack of high-throughput data to understand the mechanism of this reversible dynamic modification under biotic stresses, and low-throughput mechanistic studies cannot show the genome-wide localization of m^6^A modifications (Anderson et al., 2018). To address this issue, we used MeRIP-seq technologies to examine m^6^A methylation profiles in pear at different time points after fire blight inoculation. Our study indicated that these dynamic and reversible modifications appear to be selective. Specifically, our study revealed that m^6^A modifications can be selectively added or removed to regulate the plant defense response (Figure 5). In previous m^6^A studies in *Arabidopsis*, the effect of m^6^A on mRNA processing was mainly examined by measuring the function of m^6^A regulators (Ruzicka et al., 2017). In a recent study, the number of m^6^A writers in higher plants was found to be much greater than that in lower plants, indicating that m^6^A methylation may be more complicated and precise in higher plants (Yue et al., 2019). However, the function and existence of these regulators are still not clear. The role of the m^6^A eraser FTO in the demethylation of m^6^A has been recognized in mammals, but homologues have not been found in plants (Jia et al., 2011; Miao et al., 2020). In this context, we herein present the first MeRIP-seq analysis of pear. Overall, m^6^A methylation is highly conserved in pear, and the canonical m^6^A consensus motif RRACH and plant-specific motif URUAY are both enriched in pear (Figure 4). m^6^A modifications are mainly distributed around the start and stop codons, and the distribution does not change much after fire blight inoculation.

Almost all Rosaceae plants have undergone multiple genome duplication events; the most recent whole-genome duplication occurred in pear and apple 30–45 million years ago, but not in other Rosaceae (Wu et al., 2013). The Chinese white pear genome is approximately 512 Mb and displays 42,431 coding genes, providing a foundation to study the evolutionary mode of m^6^A in plants (Wu et al., 2013). Previous studies in mammals have shown that m^6^A is associated with selective constraints (Ma et al., 2017). A recent study reported that m^6^A modification divergence of duplicate genes can affect subgenome dominance by impacting gene expression abundance (Miao et al., 2020). However, in-depth studies of the relationship between m^6^A and gene duplication evolution are still missing. Here, we provide a new way to investigate the connection between m^6^A and duplicated gene pairs. We classified the duplicated genes by five duplication types (Qiao et al., 2019) (DSD, TD, PD, TRD, and WGD), and then the gene pairs were reclassified into non-m^6^A, single-m^6^A, and both-m^6^A to examine the coevolution of m^6^A and different duplication types. It was found that m^6^A preferentially modified duplicate genes rather than singleton genes (Figure 4F). The genes generated by WGD had the highest methylation rates, and they maintained m^6^A modification after duplication events better than other duplication types (Figure 4G). Moreover, we examined the K_a_/K_s_ values of non-m^6^A, single-m^6^A, and both-m^6^A gene pairs. K_a_/K_s_ values are usually divided into positive selection (K_a_/K_s_> 1), neutral selection (K_a_/K_s_ (=1), and purifying selection (Liu et al., 2020) (K_a_/K_s_ < 1). The m^6^A-modified genes are more conserved with smaller K_a_/K_s_ values than those without m^6^A modifications, suggesting that m^6^A-modified genes have experienced stronger purifying selection (Figure 4H and Supplementary Figures 4D–F). The association of m^6^A and gene duplication may be important. By counting the percentages of gene pairs showing K_a_/K_s_> 1, non-m^6^A genes were obviously subjected to stronger selective pressure (Figure 4I and Supplementary Figure 4G).

The functions of m^6^A genes deserve further attention. Although m^6^A is a dynamic process, the majority of m^6^A modification sites will not change much, and those small sites that change the modification sites deserve to be more precisely investigated (Figure 3A). Although there have been several studies on the transcriptome-wide map of m^6^A modifications, there are scarce studies comparing m^6^A preference and dynamic changes. Selective stabilization of salt-responsive transcripts by m^6^A has been proposed in recent reports (Anderson et al., 2018; Zheng et al., 2021); however, studies on effects following biotic stress generally focused on the role of m^6^A regulators. We used MeRIP-seq to establish the temporal m^6^A modification patterns of pear under biotic stress to examine the m^6^A change. In our study, we found that m^6^A methylation is a reversible equilibrium process. m^6^A had a preference for defense-related transcripts after inoculation, and we clearly demonstrated that the addition of m^6^A modifications can increase mRNA abundance (Figures 3B, 6E). Furthermore, we identified a considerable number of key defense-related genes, and these genes might become potential biomarkers of plant resistance. Similarly, the demethylation process of m^6^A is worth investigating, and the functions of transcripts with methylation loss were mainly related to transcriptional regulation and various biological processes. At present, it is not clear why these transcripts lost m^6^A modifications after inoculation. We can reasonably hypothesize that plants can maintain the mRNA dynamic equilibrium by regulating the m^6^A abundance of specific transcripts. Therefore, it is crucial to determine which types of defense signaling lead to the activation of m^6^A methylation and demethylation under stress.

## Conclusion

Using a combined RNA-seq and MeRIP-seq approach, we reveal an early response to fire blight in pear. The m^6^A modification patterns of pear lay the groundwork for comprehensive understanding of the m^6^A methylation and demethylation in plants. Our data highlight the importance of m^6^A in mRNA stabilization, response defense, gene duplication, and evolution. We have demonstrated that plants can regulate mRNA abundance by adding or removing m^6^A modification, and there was a significant positive correlation between mRNA abundance and m^6^A abundance. We also found that the m^6^A-modified genes experienced stronger purifying selection, further enhancing our understanding of function and evolution of m^6^A in plants.

## Data Availability Statement

The datasets presented in this study can be found in online repositories. The names of the repository/repositories and accession number(s) can be found below: https://db.cngb.org/cnsa/, CNP0001995.

## Author Contributions

SZ and XH conceived and designed the study. XH improved the methodology of data collection and analysis. QQ, CH, YZ, and FZ contributed the collecting samples. XQ was responsible for analysis and interpretation of the part of gene duplication. CH and FZ performed the bioinformatics analysis. CH wrote the article. All authors approved the submitted version of this article.

## Conflict of Interest

The authors declare that the research was conducted in the absence of any commercial or financial relationships that could be construed as a potential conflict of interest.

## Publisher’s Note

All claims expressed in this article are solely those of the authors and do not necessarily represent those of their affiliated organizations, or those of the publisher, the editors and the reviewers. Any product that may be evaluated in this article, or claim that may be made by its manufacturer, is not guaranteed or endorsed by the publisher.

## Footnotes

1
http://kobas.cbi.pku.edu.cn/

2
https://meme-suite.org/meme/doc/streme.html

3
https://github.com/qiao-xin/DupGen_finder

## Supplementary Material

Supplementary Figure 1 There were significant correlations among m^6^A writers, erasers, and readers. **(A)** Relative mRNA expression level of MTA1, ALKBH5B, and ECT9 in mock, 3 HPI, 12 HPI, and 72 HPI. **(B)** Correlation analysis of m^6^A regulators after fire blight inoculation. **(C)** The protein–protein interactions among m^6^A regulators.

Supplementary Figure 2 Circos plots of the m^6^A methylation pattern in pear. **(A)** m^6^A peak and gene density within each chromosome in pear plants at mock and 12 HPI. **(B)** Fold enrichment of m^6^A modification in three biological replicates at mock and 12 HPI.

Supplementary Figure 3 Overview of m^6^A methylation profiles in fire blight infected pear. **(A)** Percentage of total m^6^A peaks located throughout regions of mRNA transcript in 12 HPI. **(B)** The localization of m^6^A peaks in mock and 12 HPI pear seedlings. **(C)** Proportions of different m^6^A peak numbers in m^6^A-modified transcripts in 12 HPI pear seedlings. Error bars represent the SD of three biological repeats.

Supplementary Figure 4 Evidence for coevolution of m^6^A modifications and gene duplication. **(A)** Comparison of ratios of m^6^A gene pairs and non-m^6^A gene pairs in five duplication types. **(B)** Comparison of ratios of both-m^6^A gene pairs and single-m^6^A gene pairs in five duplication types. **(C–F)** K_a_/K_s_ ratio of non-m^6^A gene pairs (blue box), single gene pairs (orange box), and both-m^6^A gene pairs (red box) in five duplication types. **(G)** The percentages of gene pairs showing K_a_/K_s_> 1 in TRD and DSD. **(H–K)** Expression divergence among duplicate genes derived from non-m^6^A, single-m^6^A, and both-m^6^A in PD, TRD, DSD, and TD.

Supplementary Figure 5 LC-MS/MS quantification of RNA modification in mock and 12 HPI plants.

Supplementary Figure 6 qRT-PCR and m^6^A-IP-qPCR assay of m^6^A-modified genes. **(A)** m^6^A-IP-qPCR validation of m^6^A methylation level of 10 randomly selected genes from Supplementary Table 6. **(B)** Relative mRNA levels. **p* < 0.05; ^**^*p* < 0.01.
