# Supplementary figures and images for: Multi-Omics Analysis Reveals the Dynamic Changes of RNA N6-Methyladenosine in Pear (Pyrus bretschneideri) Defense Responses to Erwinia amylovora Pathogen Infection
Source: Front Microbiol. 2022 Feb 10;12:803512. doi: 10.3389/fmicb.2021.803512 (PMC8867029; doi:10.3389/fmicb.2021.803512)

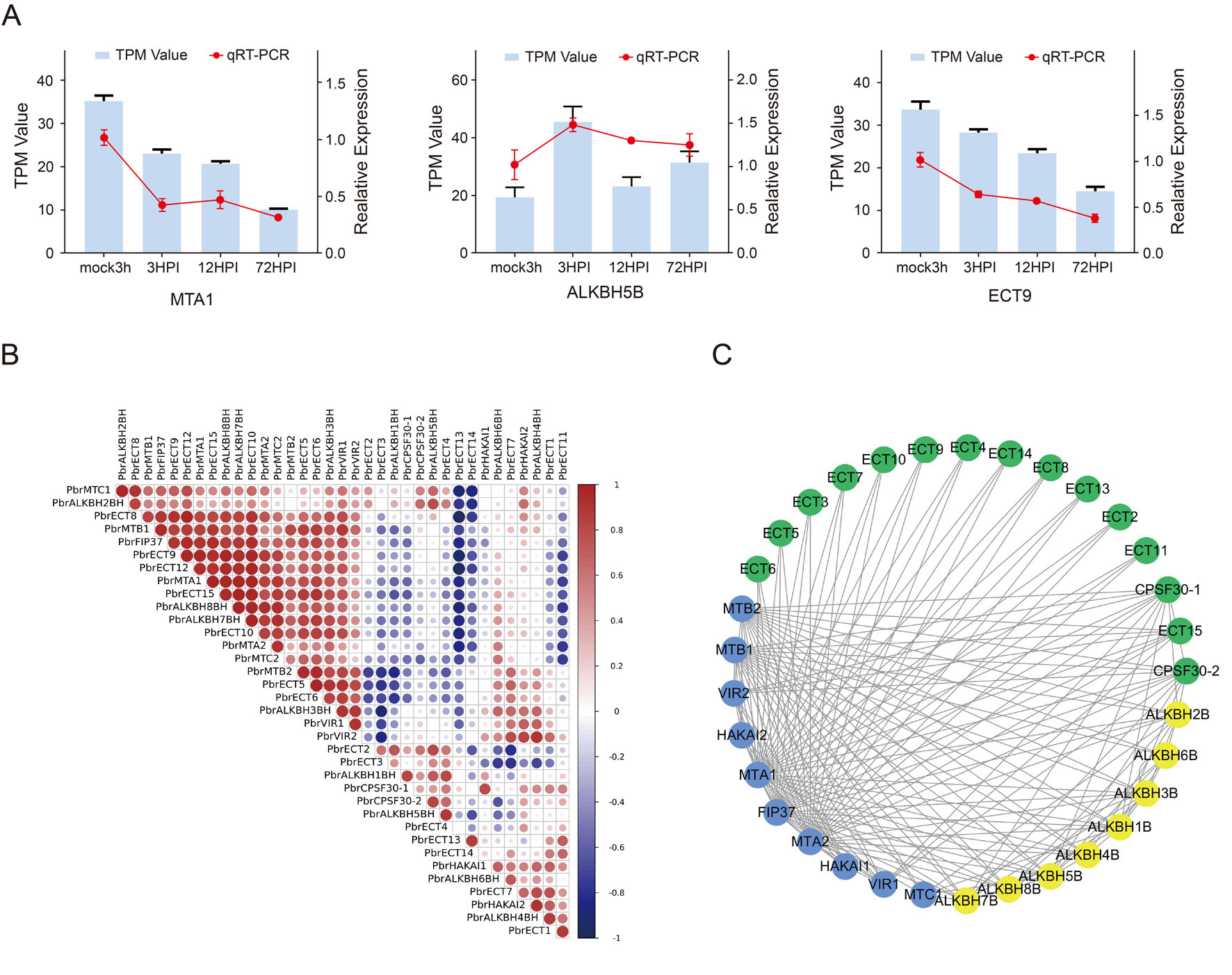

Supplement: Supplementary Figure 1 — There were significant correlations among m6A writers, erasers, and readers. (A) Relative mRNA expression level of MTA1, ALKBH5B, and ECT9 in mock, 3 HPI, 12 HPI, and 72 HPI. (B) Correlation analysis of m6A regulators after fire blight inoculation. (C) The protein–protein interactions among m6A regulators. [file Image_1.jpeg]

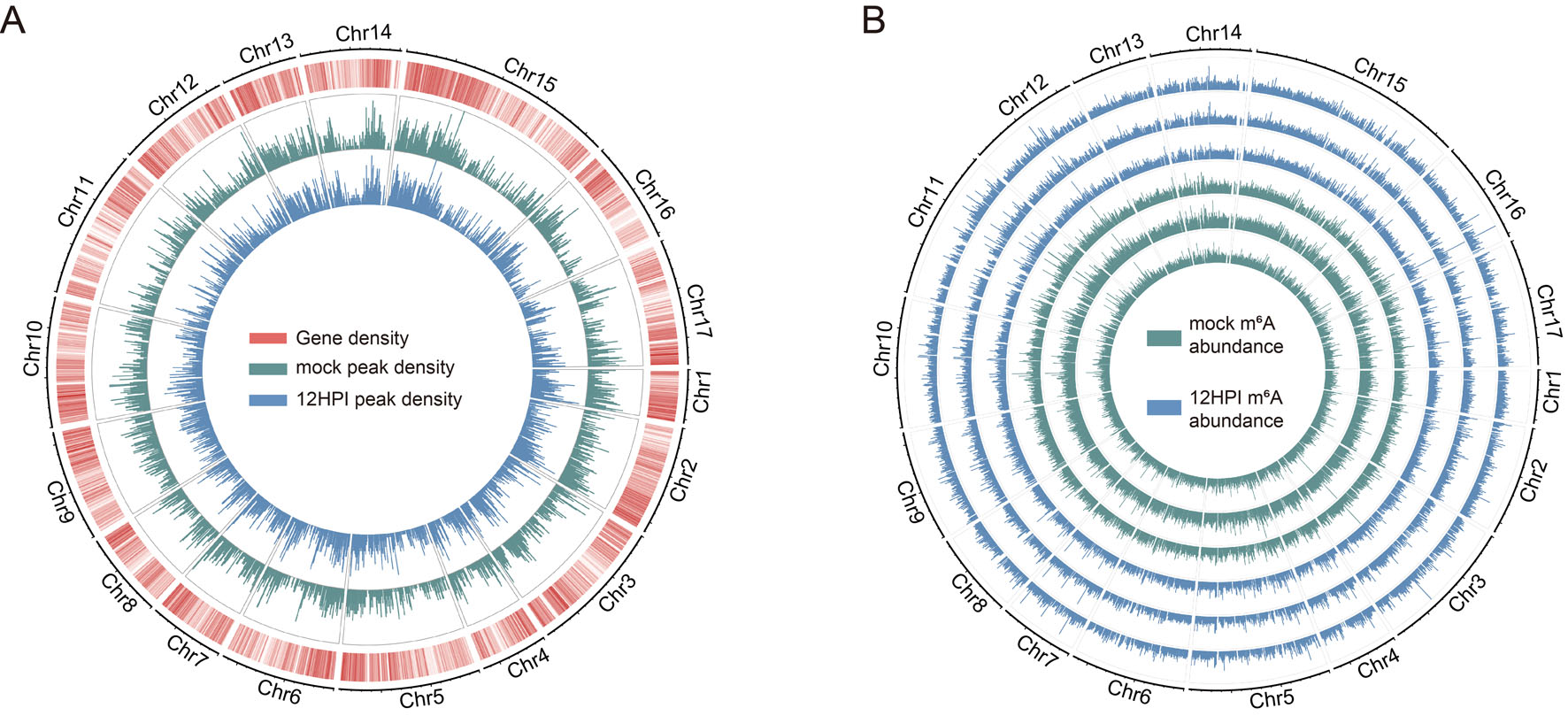

Supplement: Supplementary Figure 2 — Circos plots of the m6A methylation pattern in pear. (A) m6A peak and gene density within each chromosome in pear plants at mock and 12 HPI. (B) Fold enrichment of m6A modification in three biological replicates at mock and 12 HPI. [file Image_2.jpeg]

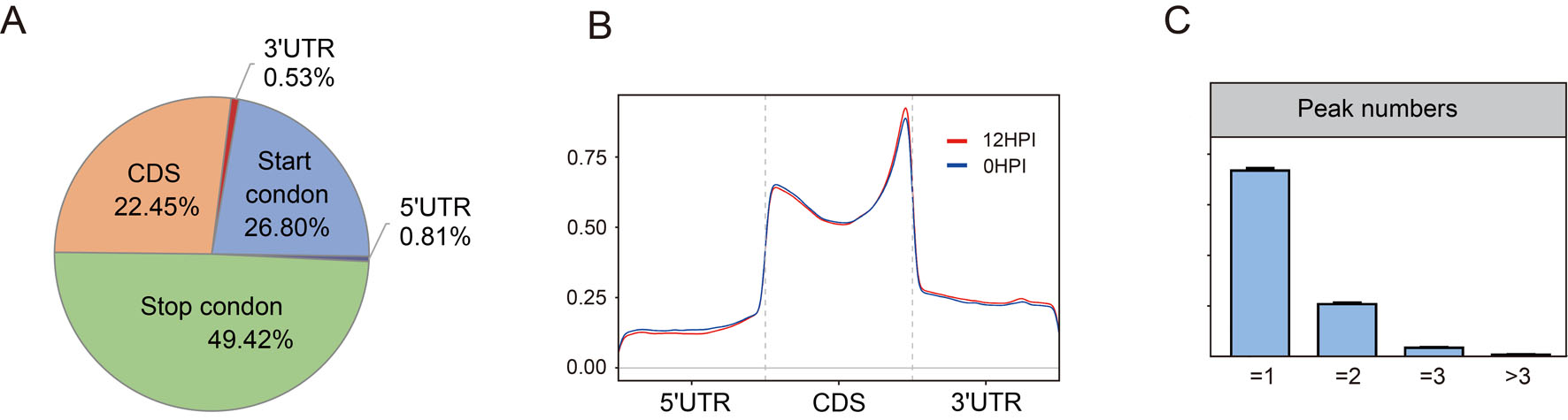

Supplement: Supplementary Figure 3 — Overview of m6A methylation profiles in fire blight infected pear. (A) Percentage of total m6A peaks located throughout regions of mRNA transcript in 12 HPI. (B) The localization of m6A peaks in mock and 12 HPI pear seedlings. (C) Proportions of different m6A peak numbers in m6A-modified transcripts in 12 HPI pear seedlings. Error bars represent the SD of three biological repeats. [file Image_3.jpeg]

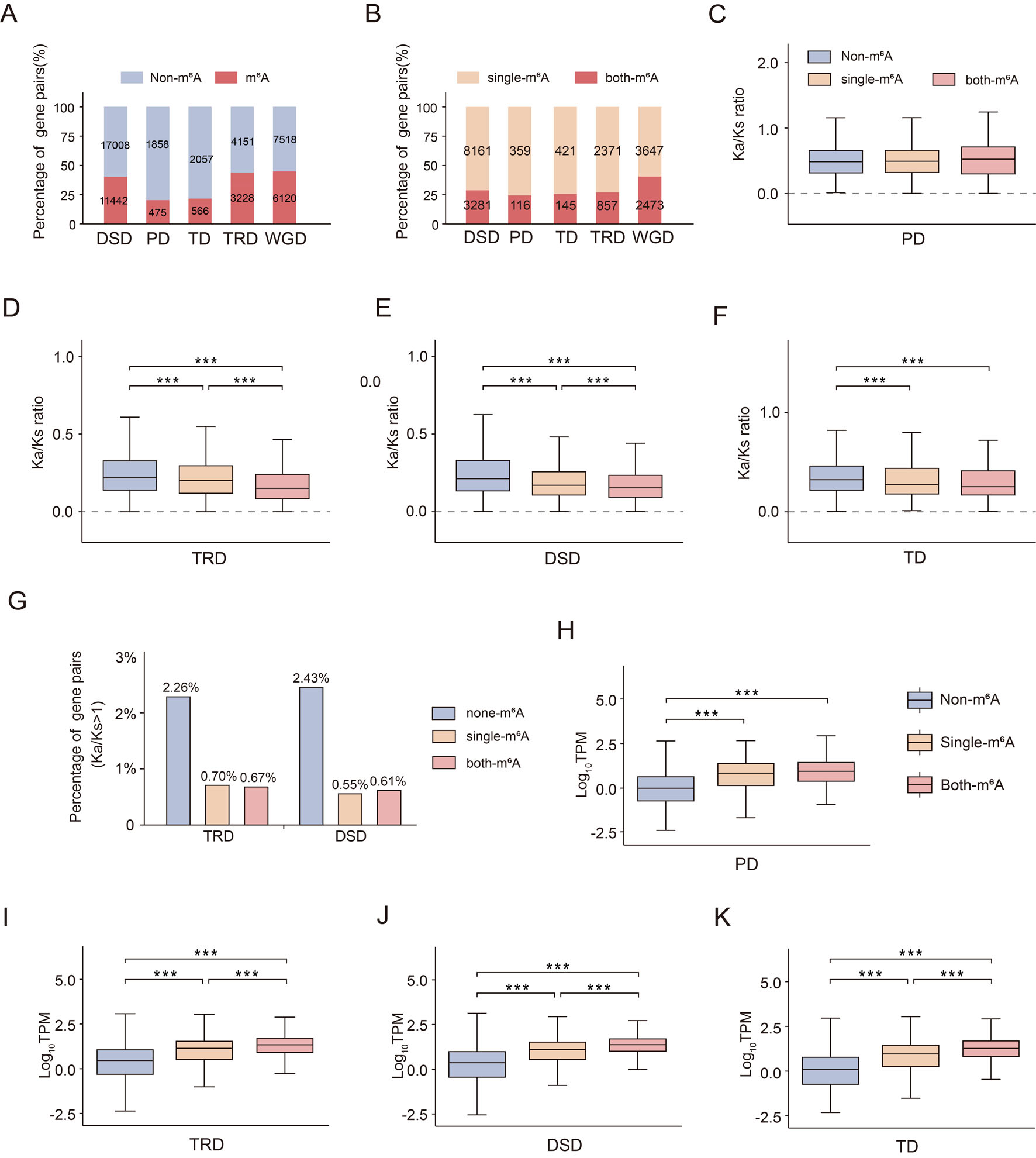

Supplement: Supplementary Figure 4 — Evidence for coevolution of m6A modifications and gene duplication. (A) Comparison of ratios of m6A gene pairs and non-m6A gene pairs in five duplication types. (B) Comparison of ratios of both-m6A gene pairs and single-m6A gene pairs in five duplication types. (C–F) Ka/Ks ratio of non-m6A gene pairs (blue box), single gene pairs (orange box), and both-m6A gene pairs (red box) in five duplication types. (G) The percentages of gene pairs showing Ka/Ks> 1 in TRD and DSD. (H–K) Expression divergence among duplicate genes derived from non-m6A, single-m6A, and both-m6A in PD, TRD, DSD, and TD. [file Image_4.jpeg]

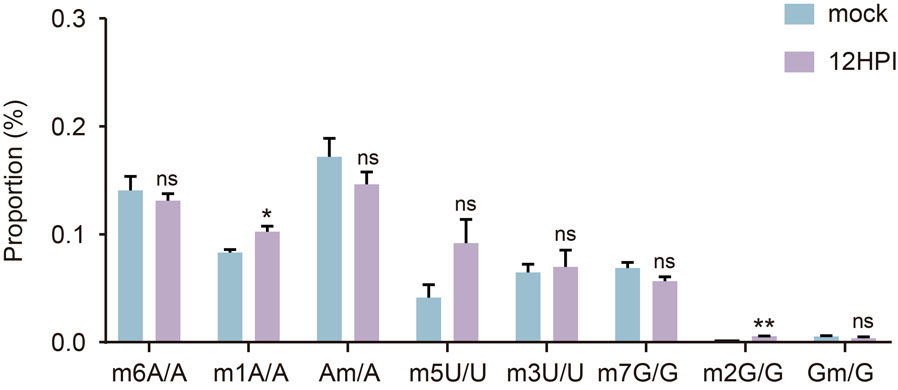

Supplement: Supplementary Figure 5 — LC-MS/MS quantification of RNA modification in mock and 12 HPI plants. [file Image_5.jpeg]

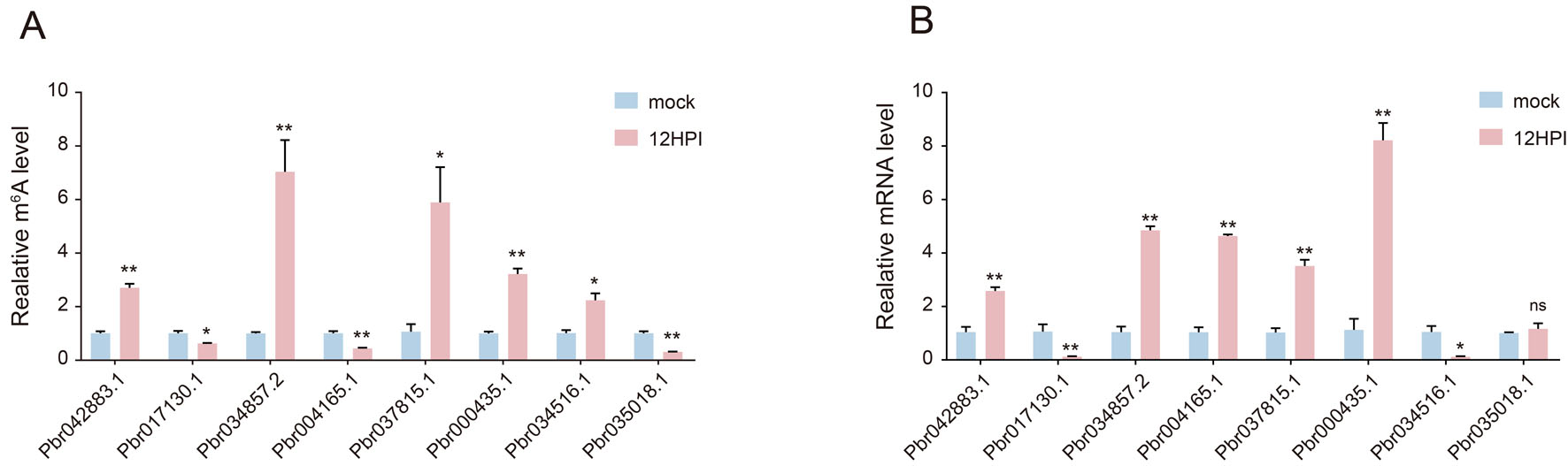

Supplement: Supplementary Figure 6 — qRT-PCR and m6A-IP-qPCR assay of m6A-modified genes. (A) m6A-IP-qPCR validation of m6A methylation level of 10 randomly selected genes from Supplementary Table 6. (B) Relative mRNA levels. *p < 0.05; **p < 0.01. [file Image_6.jpeg]
